# Corrigendum to “Assessments and interventions in individuals with lower extremity torsional abnormality: A scoping review” [J Orthopedics 73 (2026) 247–261]

**DOI:** 10.1016/j.jor.2026.02.030

**Published:** 2026-02-06

**Authors:** M. Gagnon, N. Abdel Fattah, L. Groszman, N. Kabbes, M. Bernstein, L.N. Veilleux

**Affiliations:** a Department of Surgery, McGill University, Montreal, QC, Canada; b Motion Analysis Center, Shriners Hospitals for Children-Canada, Montreal, QC, Canada; c Faculty of Medicine and Health Sciences, McGill University, Montreal, QC, Canada; d Division of Orthopaedic Surgery, University of Toronto, Toronto, ON, Canada; e Limb Deformity Unit, Shriners Hospitals for Children, Montreal, QC, Canada

**Keywords:** Lower Extremity torsional abnormality, Femoral anteversion, Tibial torsion, Assessment, Surgery, Derotational osteotomy, Conservative management, Pediatric orthopaedic

## Abstract

**Introduction:**

Lower extremity torsional abnormalities (LETA) are a frequent reason for referral to pediatric orthopaedic surgeons. Defined as abnormal tibial or femoral torsion along the longitudinal axis, they affect gait biomechanics and may cause functional, cosmetic, and psychosocial concerns. Despite their prevalence, assessment methods and management strategies remain heterogenous, and thresholds for intervention are unclear.

**Methods:**

A scoping review was conducted following Arksey and O'Malley framework and the PRISMA-ScR guideline to map the current evidence regarding assessments and interventions. MEDLINE, Embase, and Cochrane were searched (2000–2025) for English and French studies involving youth (0-21 years) with idiopathic LETA. Eligible designs included randomized trials, cohort studies, case series, and cross-sectional studies. Data were extracted, synthesized descriptively, and organized by torsion type, assessment modality, and intervention strategy.

**Results:**

Of 11,174 records identified, 56 studies met inclusion criteria. Most were retrospective (n = 35) and conducted in North America and Europe. Assessment methods included physical examination (e.g., hip rotation, thigh–foot angle), imaging (CT, MRI, biplanar radiographs), and quantitative motion analysis. Management strategies ranged from observation and orthotics to surgical derotational osteotomies. Considerable variation existed in measurement techniques, intervention thresholds, and outcome reporting, with limited high-level evidence.

**Conclusion:**

This scoping review highlights variability in how LETA are assessed and managed. Standardized assessment protocols, clearer reporting of surgical indications, and systematic evaluation of conservative management remain key gaps. Addressing these areas would strengthen the evidence base and guide clinical decision-making.

The authors regret that the abstract was omitted from the published version of this article. The abstract is provided below.

The authors would like to apologise for any inconvenience caused.

